# Rheumatoid Arthritis Fibroblast-like Synoviocyte Suppression Mediated by PTEN Involves Survivin Gene Silencing

**DOI:** 10.1038/s41598-017-00517-w

**Published:** 2017-03-23

**Authors:** Danna Chen, Dongdong Liu, Dan Liu, Min He, Anping Peng, Jiarui Xu, Li Lin, Fudong Luo, Lin Chen, Xianzhang Huang, Junhua Zhuang, Jianhua Xu

**Affiliations:** 1 Department of Laboratory Science, Zengcheng District People’s Hospital of Guangzhou (BoJi-Affiliated Hospital of Sun Yat-sen University), Guangzhou, 511300 China; 2 Department of Laboratory Science, the Second Affiliated Hospital of Guangzhou University of Chinese Medicine, Guangzhou, 510120 China

## Abstract

Survivin is a proto-oncogene biomarker known for its anti-apoptotic and cell cycle regulating properties induced by the activation of the phosphoinositide 3-kinase (PI3K)/Akt pathway. In the context of non-cancer pathology, such as rheumatoid arthritis (RA), survivin has emerged as a feature associated with severe joint damage and poor treatment response. Phosphatase and tensin homolog (PTEN) is a phosphatase antagonizing all classes of PI3K. The interplay between survivin oncogenic mechanisms and proliferation suppression networks in RA has remained largely elusive. This study investigated the effect of PTEN on survivin gene expression in rheumatiod arthritis fibroblast-like synoviocyte (RA-FLS). We showed for the first time that the suppression of RA-FLS was mediated by PTEN involving survivin silencing. Considering that survivin suppressants are currently available in clinical trials and clinical use, their effects in RA-FLS support a probably RA therapy to clinical practice.

## Introduction

Rheumatoid arthritis (RA) is a progressive debilitating autoimmune disease leading to cartilage and bone destruction caused by insufficient apoptosis in the inflamed RA synovium. It affects 1% of the world population, and recent treatment has been revolutionized by the use of biologic therapies, such as drugs that target cytokines, cells, and signaling pathways. However, half of RA patients display drug resistance to biological therapies, which leads to early mortality and demonstrates our limited knowledge of RA pathogenesis^1^.

Survivin is the smallest member of the inhibitor of apoptosis protein (IAP) family and it is encoded by the BIRC5 gene^2^. It has recently emerged as a biomarker of RA. High levels of survivin are detected in the blood and synovial fluid of patients with RA and are associated with early joint damage and poor therapy response^3, 4^. Functionally, the protein has abilities to inhibit apoptosis, promote cell proliferation, and produce cytokines and growth factors. Additionally, survivin was shown to promote the transformation of synovial fibroblasts to an invasive phenotype, followed by the proliferation of synovial tissue and pannus formation^5^. Clinical and experimental findings indicate a key role of survivin in the pathogenesis of RA.

The phosphatidylinositol 3-kinase (PI3K) pathway is one of the most important signal transduction pathways involved in the regulation of fundamental processes, such as cell survival, cell migration, proliferation and cytoskeleton remodeling^6–8^, as well as leukocyte activation and immune cell homeostasis^9, 10^. Phosphatase and tensin homolog (PTEN) is an antagonizing phosphatase for all classes of PI3K^6, 11^. Studies suggest that the PI3K/PTEN axis controls multiple aspects in the pathogenesis of inflammatory diseases such as cell migration, invasive behavior, cytokine production and proliferation, and T cell polarization^12–15^.

Recent reports showed that survivin is a direct target gene of the PI3K/AKT signaling pathway in the ectopic endometrium and invasive epithelium cells in adenomyosis^16–18^. In this study, we determined whether PTEN effects survivin gene expression in rheumatiod arthritis fibroblast-like synoviocyte (RA-FLS).

## Results

### Survivin is suppressed after inhibition with LY294002 in RA-FLS

**Figure 1:**
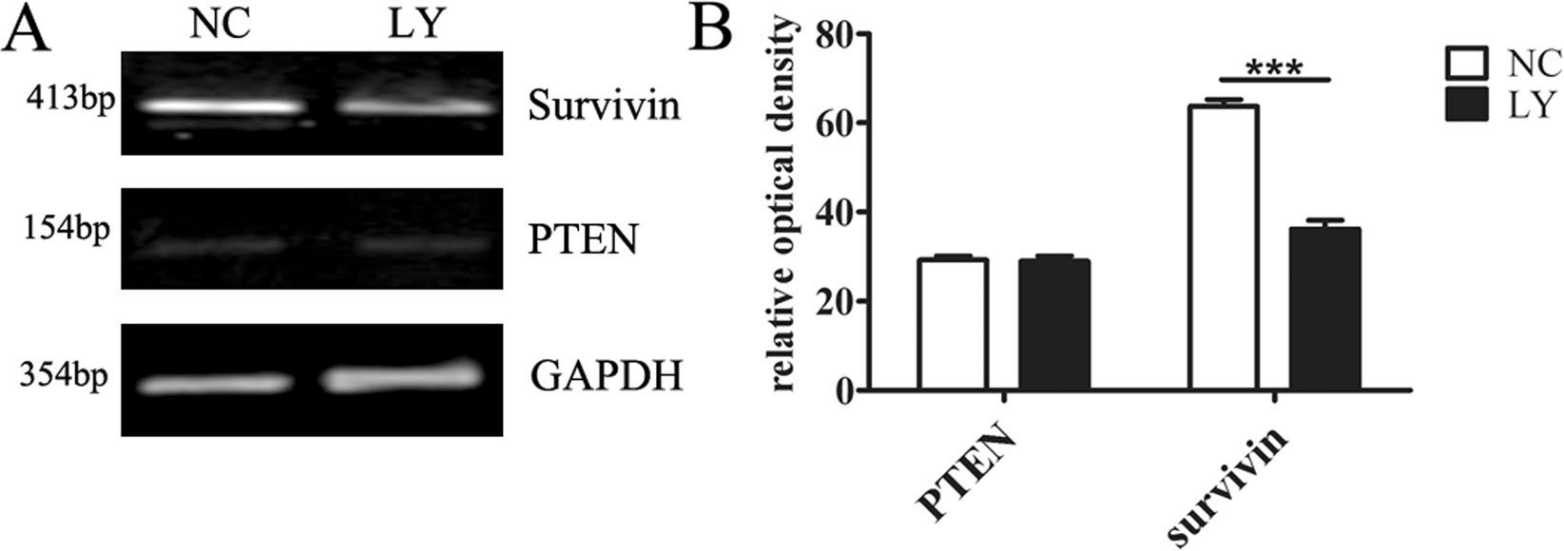
(**A**) The gene expression of survivin was suppressed by the PI3K/Akt pathway inhibitory LY294002. Meanwhile, the PTEN expression was not affected. The direct qPCR was normalized by GAPDH. (**B**) Results of triplicate experiments. The relative optical density of survivin was significantly reduced in comparison to the normal control. “***” stands for P < 0.001.

Recent reports showed that survivin is a direct target gene of the PI3K/AKT signaling pathway in the ectopic endometrium and invasive epithelium cells in adenomyosis^16–18^. In our study, we further confirmed the regulation of survivin expression in RA-FLS. As shown in Fig. 1, the mRNA levels of survivin were significantly down-regulated after inhibition of the PI3K/Akt pathway with LY294002. By contrast, expression of the PTEN gene did not significantly change in the presence of LY294002. We hypothesized that survivin expression is regulated by the PI3K/Akt pathway in RA-FLS. All evaluations were tested by directed qPCR with normalization to the housekeeping gene GAPDH (Fig. 1A). Relative expression was quantitated using densitometry, and the results of triplicate experiments are shown in Fig. 1B.

### Construction of recombinant pcDNA3.1-PTEN and shRNA-pENTR/U6-survivin

**Figure 2:**
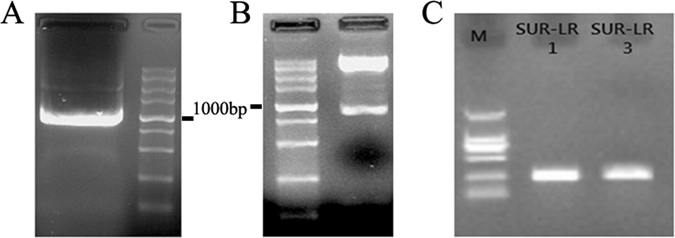
(**A**) The PCR products for PTEN were visualized under a UV transilluminator. The molecule weight (MW) was correct for PTEN (1212 bp). (**B**) The construction of pcDNA3.1-PTEN was proved correct by enzyme digestion. (**C**) Two constructs of survivin (389 bp) shRNA was verified by PCR from engineering bacteria. The DNA marker was DL5000 from Takara Company.

After electrophoresis using 1.5% agarose gel, the PCR product was visualized under a UV transilluminator and purified with the PCR product purification kit (OMEGA). The expected 1212 bp PTEN PCR product fragment was observed (Fig. 2A). The PCR product was ligated into the pcDNA3.1 expression vector and transformed into the *E. coli* DH5*α* strain. The recombinant plasmid was confirmed by restriction enzyme analysis (Fig. 2B) and sequencing analysis.

Three annealed double-stranded shRNAs were individually cloned into the linearized shuttle vector shRNA-pENTR/U6, which was verified by PCR using the U6 and V5 universal primers. Only #1 and #3 were successfully constructed (Fig. 2C). Sequencing was performed to confirm successful cloning of the recombinant shuttle plasmid.

### Over-expression of PTEN reduces the survivin expression

**Figure 3:**
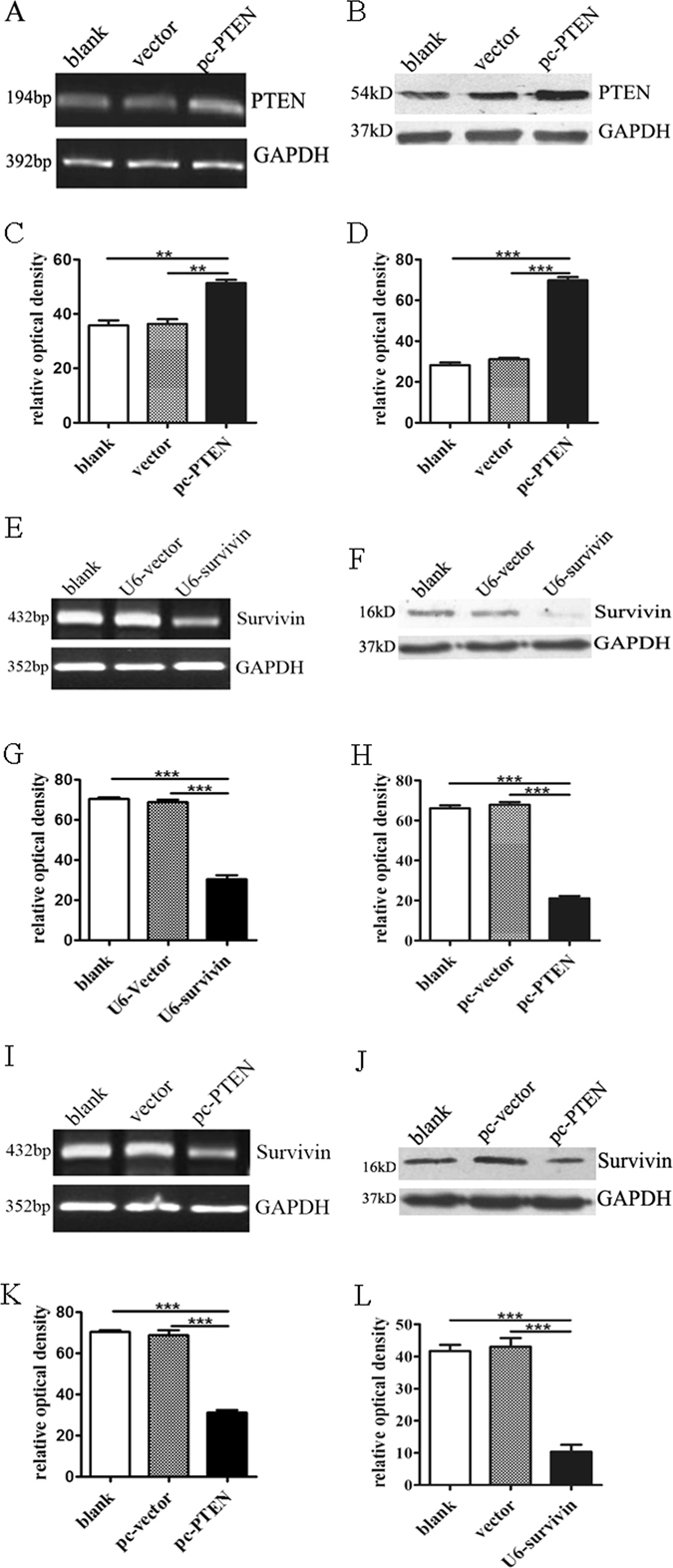
(**A** and **B**) The over-expression of PTEN in mRNA and protein. The group of blank and vector transfection were controls. (**E** and **F**) The knock-down of survivin by shRNA in RA-FLS mRNA and protein. (**I** and **J**) The down-regulation of survivin by PTEN in RA-FLS mRNA and protein. All the results were normalized to the housekeeping gene GAPDH. (**C**,**D**,**G**,**H**,**K** and **L**) The results of three times with statistical analysis. “**” stands for P < 0.01. “***” stands for P < 0.001.

The over-expression of PTEN by transient transfection of the PTEN-pcDNA3.1 plasmid in RA-FLS was verified by qPCR (Fig. 3A) and Western blotting (Fig. 3B). Blank and pcDNA3.1 vector transfection were performed as negative controls. Statistical analysis of three parallel experiments showed the efficiency and stability of gene overexpression (Fig. 3C and D). The establishment of survivin-depletion RA-FLS cells by specific shRNA was confirmed by qPCR (Fig. 3E) and Western blotting (Fig. 3F) with statistical analysis of three parallel experiments (Fig. 3G and H).

To determine whether survivin production is regulated by the PI3K/Akt pathway inhibitor PTEN, RA-FLS cells transfected with a PTEN over-expression plasmid or control were measured the levels of mRNA and protein of survivin after 24 hours transfection. A significant reduction of survivin production was observed by qPCR (Fig. 3I) and Western blotting (Fig. 3J). This inhibitory effect was not observed in cells transfected with control plasmid or blank. Three parallel experiments showed similar results with significant differences (*P* < 0.001) (Fig. 3K and L). All results suggested that the up-regulation of PTEN can decrease survivin gene expression. We hypothesized that the inhibition of PTEN on the PI3K/Akt pathway leading to the down-regulation of survivin. The result was similar to the research in cells in adenomyosis^16–18^.

### PTEN over-expression and/or silencing of survivin inhibited RA-FLS proliferation

**Figure 4:**
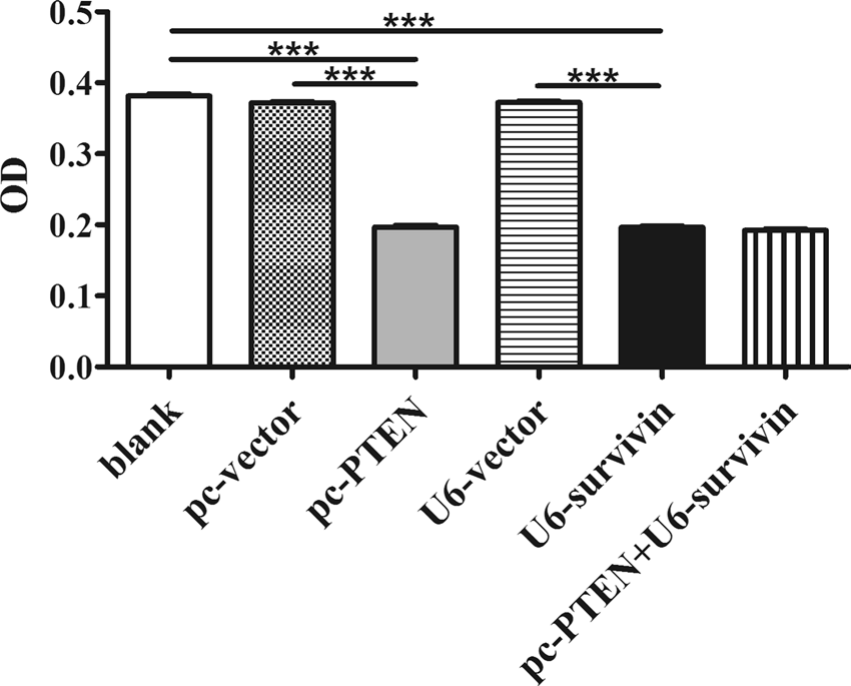
CCK-8 test for cell proliferation. The proliferation rate was significantly reduced in PTEN over-expression and survivin down-regulated RA-FLS. But no more reduction was found in co-transfection RA-FLS. “***” stands for P < 0.001.

The regulation of cell division is a prominent function of survivin. The stimulatory function on RA-FLS proliferation by survivin is important in disease development^5^. We verified the significance of survivin on RA-FLS proliferation using CCK8 tests. RA-FLS cells with survivin knock-down showed an inhibition of cell proliferation (Fig. 4). The lipid phosphatase PTEN is a well established inhibitor of cell proliferation. In our study, a similar reduction in cell growth was exhibited in the PTEN over-expression group.

To determine the relationship between PTEN over-expression and survivin knock-down, co-transfection of PTEN-pcDNA3.1 and survivin shRNA was performed simultaneously. The co-transfection did not show any cooperative relationship on RA-FLS cell proliferation inhibition. There was no significant change between the co-transfection group and either transfection group alone (Fig. 4).

### Migration is decreased by PTEN over-expression or survivin knock-down

**Figure 5:**
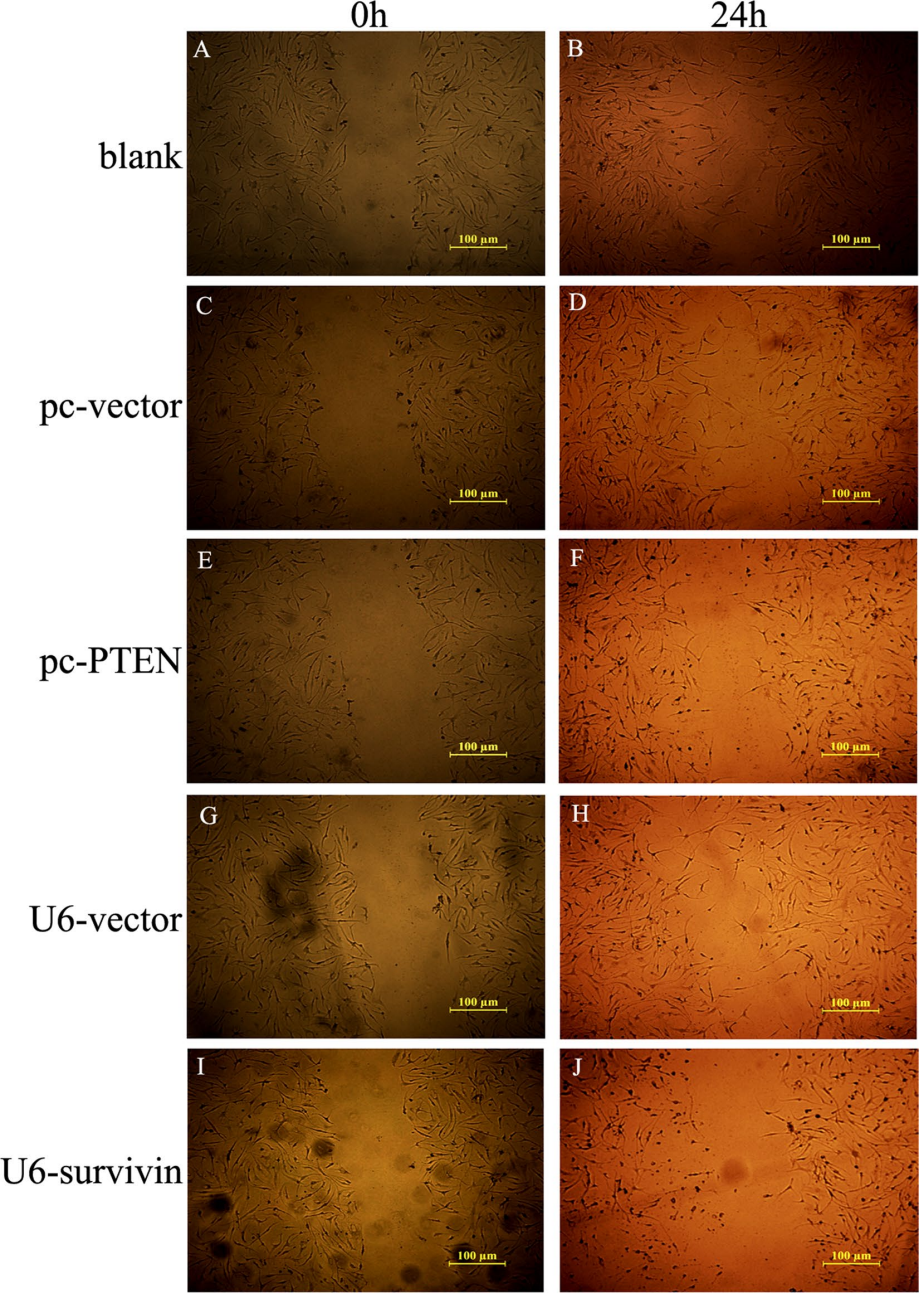
Representative images of cell density in wound-healing assay. (**A**,**C**,**E**,**G** and **I**) were representive pictures of wound healing assay at 0 h. (**B**,**D**,**F**,**H** and **J**) were representive pictures of wound healing assay after 24 h. (**A** and **B**) Showed the RA-FLS of blank. (**C** and **D**) Showed the RA-FLS of pc-vector control transfection. (**E** and **F**) Showed the RA-FLS with pc-PTEN over-expression. (**G** and **H**) Showed the RA-FLS of U6-vector control transfection. (**I** and **J**) Showed the RA-FLS with survivin down-regulation.

Wound healing assays are used for analysis of cell migration *in vitro*. As shown in Fig. 5, the effect of the PTEN over-expression or survivin knock-down on RA-FLS cells migration was detected to be negative compared to the normal control. We propose that PTEN over-expression or survivin knock-down impairs the migration of RA-FLS.

### The effect of PTEN and survivin on arthritis inflammation and RA-FLS invasion

**Figure 6:**
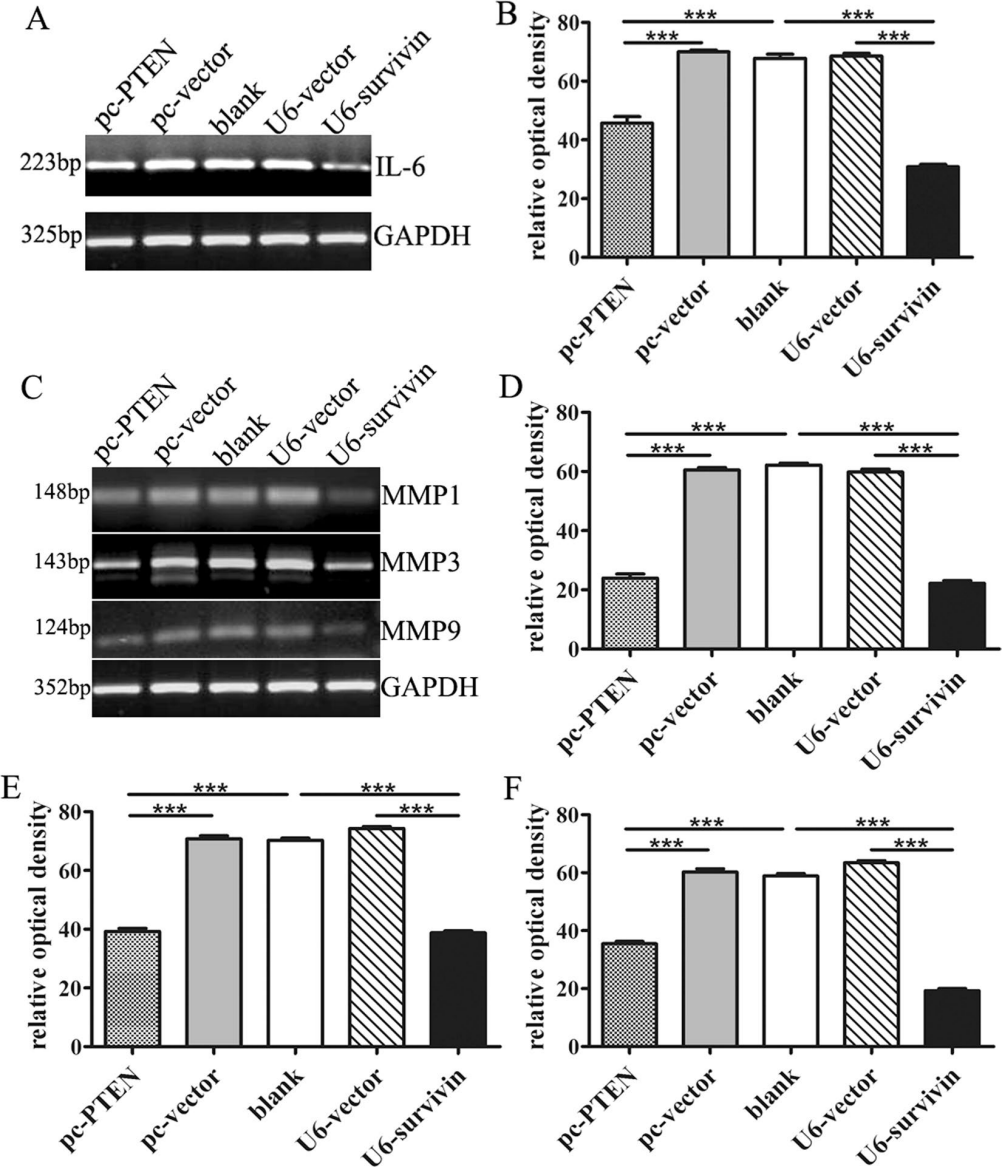
The inflammation cytokine IL-6 (**A**) and invasion molecules MMP1, MMP3 and MMP9 (**C**) were down-regulated by PTEN over-expression and survivin knock-down in the direct qPCR results with internal control of GAPDH. The change of relative optical density in three parallel experiments of IL-6 (**B**), MMP1 (**D**), MMP3 (**E**) and MMP9 (**F**) were analyzed to be significant (P < 0.001).

The pro-inflammatory cytokine IL-6 is important for potentiating the inflammatory response in RA^15^. In our study, the expression of IL-6 was reduced by PTEN over-expression or survivin knock-down. The results suggest the important role of PTEN and survivin in RA inflammation (Fig. 6A).

The matrix metalloproteinases (MMPs), such as MMP1, MMP3 and MMP9 may be important in the destructive joint changes of RA^19, 20^. PTEN also inhibits RA-FLS invasion. MMPs were significantly down-regulated in the presence of PTEN over-expression and survivin silencing (Fig. 6C). The triplicate results with statistical analysis were shown in Fig. 6B,D,E and F.

## Discussion

This study shows a close relationship between PTEN and survivin for nearly all RA-FLS functional processes that induce the pathogenesis of RA, such as cell proliferation, migration, invasion and inflammation.

Survivin is a direct target gene of the PI3K/AKT signaling pathway in the ectopic endometrium and invasive epithelium cells in adenomyosis^16–18^. In our study, we showed the regulation of this pathway in RA-FLS. The PI3K inhibitor LY294002, significantly decreased the expression of survivin. Then, we explored the relationships between PTEN, the antagonizing phosphatase of PI3K/AKT pathway, and survivin by the construction and transfection of plasmids that over-express PTEN and knock-down survivin in RA-FLS. Consistent with the results of the LY294002 inhibitor, PTEN overexpression reduced survivin gene-expression. The results provide direct evidence of the survivin regulation by the PTEN/PI3K/AKT pathway in RA-FLS. The hyporhesis was similar to the research in adenomyosis^16–18^. So we got a general idea that the PTEN/PI3K/AKT pathway on survivin expression seemed to be the molecular mechanism underlined in the epithelium cell biological malignant behavior.

The close associations between survivin and RA disease activity, production of autoantibodies, and the predicted development of joint damage^3, 21^ were previously reported. In our study, we also found that survivin is important in RA. The results showed that survivin helps the tumor-like proliferation of RA-FLS and is involved in the secretion of the proinflammatory cytokine IL-6 and MMPs. IL-6 is one of the most abundantly expressed cytokines identified in the sera of RA patients. It promotes B cell growth and differentiation, Th17 cell generation, and osteoclast formation^22–25^. The levels of MMP-1 and MMP-3 were proved to correlated with the degree of articular surface erosion^26^. MMP-9 was showed to involve in the angiogenesis and the pannus formation in RA synovium tissues, which has been considered as essential events in the development of RA^27^. All the previous studies, as well as our results elucidated the molecules play pivotal roles in the development of synovial hyperplasia, sustained inflammation and joint destruction in arthritic joints.

In our study, we showed the important role of PTEN in RA. We provide an integrated explanation for the down-regulation of PTEN initially shown by the Thomas Pap group^13^. The loss of PTEN promotes RA-FLS in cell proliferation, migration, invasion and inflammation.

Notably, the simultaneous overexpression of PTEN and silencing of survivin did not have a greater effect on inhibition of cell proliferation. We hypothesize that PTEN may play the inhibitory roles by targeting survivin, and that survivin can only be reduced by the PTEN/PI3K/AKT pathway in RA-FLS cell proliferation.

## Methods

### Ethics approval

This experimental protocols and consent procedure was approved by the ethics committee of the Second Affiliated Hospital of Guangzhou University of Chinese Medicine, Guangdong, China. In addition, all the participants were given written informed consent in the study. All procedures and methods involving human samples were in accordance with approved guidelines.

### RNA isolation and reverse transcription polymerase chain Reaction

Total RNA was isolated by TRIzol reagent (Life Technologies, Basel, Switzerland) according to the manufacturer’s protocol. First strand cDNA was synthesized by oligo(dT) primers and Moloney murine leukemia virus (M-MuLV) reverse transcriptase (Boehringer-Mannheim, La Jolla, California, USA).

### Plasmid construction

A 1212 base pair PTEN cDNA fragment was amplified using polymerase chain reaction (PCR) with primers of fwd: 5′-ATAGGATCCATGACAGCCATCATCAAAGAG-3′ and rev: 5′-CCGCTCGAGTTTCATGGTGTTTTATCCCTC-3′ with KOD-Plus-DNA-polymerase (Toyobo Co., Ltd., Japan) from cDNA of peripheral blood mononuclear cells, digested with *BamH*/*Xho* I and cloned into pcDNA3.1 vector. Short hairpin RNA (shRNA)-pENTR/U6 entry vector of survivin was constructed with top strand of 5′-CACCGCAAAGGAAACCAACAATACGAATATTGTTGGTTTCCTTTGC-3′ and bottom strand of 5′-AAAAGCAAAGGAAACCAACAATAT TCGTATTGTTGGTTTCCTTTGC-3′ selected from Invitrogen shRNA analysis software and BLAST analysis. Primers of negative control were fwd: 5′-CACCGT CACAACCTCCTAGAAAGAGTAGACGAATCTACTCTTTCTAGGAGGTTGTGA-3′ and rev: 5′-AAAATCACAACCTCCTAGAAAGAGTAGATTCGTCTACTCTT TCTAGGAGGTTGTGAC-3′. Then survivin pENTR/U6 entry plasmid and pLenti6/BLOCK-iT lentiviral expression plasmid were used to carry out the LR recombined reaction. After plasmids were extracted, U6 upstream primer and V5 downstream primer were used to carry out PCR for identification.

### Tissue preparation and cell cultures

Synovial tissue specimens were obtained from five RA patients (F = 3, M = 2, average age = 55.8 ± 9.7) undergoing synovectomy or joint replacement in the Second Affiliated Hospital of Guangzhou University of Traditional Chinese Medicine and the Third Affiliated Hospital of Sun Yat-sen University. All the patients were diagnosed by the 1987 American College of Rheumatology criteria for RA^28^ and had actively inflamed knee joints. Immediately after surgery, the tissues were brought to our laboratory and enzymatically digested. The released cells were grown in Dulbecco’s modified Eagle medium (DMEM) with 10% fetal bovine serum (FBS) in a humidified incubator maintained at 37 °C in the presence of 5% CO_2_^29^. Cells from passages 4–6 were used in the experiments. The results have been collected in all five patients, and the results were similar in all five patients.

In experiments assessing the role of PI3K inhibition on survivin, 100 μM LY294002 (Cell Signaling Technology, Beverly, MA, USA) was added to culture media and DMSO as a control.

### Transient Transfection

For *in vitro* transfections of plasmids, cells were seeded on 6-well plates at a density of 1 × 10^5^ cells per well in medium without antibiotics and cultured until 70 to 80% confluent. Before transfection, we optimized the commercial transfection reagents of Opti-MEM | Reduced Serum Medium (Invitrogen Life Technologies, Inc., Carlsbad, CA, USA) and Lipofectamine 2000 (Invitrogen) according to the manufacturer’s instructions. The control groups were transfected without plasmid or with pcDNA3.1/U6 vector. After 6 h transfection, we changed the medium with 10% FBS DMEM medium and cultivated to 24 h. Then cells were collected to analyze protein and RNA expression by Western blot analysis and qRT- PCR.

### Direct qPCR

We utilized the direct qPCR to evaluate the gene expression with 2 × master mixes (New England BioLabs, USA) and specific primers. Primers used were: PTEN fwd: CCAGTCAGAGGCGCTATGTG; PTEN rev: TAGCTGGCAGACCACAAACTG; Survivin fwd: ATGGGTGCCCCGACGTTG; Survivin rev: ACTCTGGGACCAGGCAGCT; MMP-1 fwd: CCTGGAGGAAATCTT GCTCATGCT; MMP-1 rev: GTCCAAGAGAATGGCCGAGTTCA; MMP-3 fwd: CACCAGCATGAACCTTGTTCAGA; MMP-3 rev: TCACCTCCAATCCAAGG AACTTCT; MMP-9 fwd: CACCTCG AACTTTGACAGCGA; MMP-9 rev: CTCCGGCACTGAGGAATGATCT; IL-6 fwd: GTCCAGTTGCCTTCTCCC; IL-6 rev: GCCTCTTTGCTGCTTTCA; GAPDH fwd: GGGGCCATCCACAGTCTTC; GAPDH rev: CACCATCTTCCAGGAGCGAG. The qPCR products were examined by agarose gel electrophoresis.

### Western blotting analysis

Cells were lysed at 4 °C with RIPA lysis buffer containing 1 μmol/L PMSF. Equal amounts of protein were separated by sodium dodecyl sulphate-polyacrylamide gel electrophoresis and electrotransferred onto polyvinylidene fluoride membrane. The membrane was sealed with PBS containing 5% Skimmed milk powder for 1 h and incubated with primary antibodies (survivin, PTEN and GAPDH) at 4 °C overnight. The membrane was washed with PBST (PBS, pH 7.5, containing 0.1% Tween-20) and incubated with a secondary antibody for 1 h. Anti-mouse or anti-rabbit antibodies against IgG conjugated with HRP were adopted as the secondary antibodies. Immobilon western HRP substrate was used to detect peroxidase activity.

### Wound healing assay

In wound healing assay, the cells were cultured to 80–90% confluence in six-well plates. A 200 μl sterile pipet tip was used to make a central linear wound, and the plates were washed with PBS to remove the floating cells. The speed of wound closure was analyzed by measuring the distance of the wound edge to the original wound site after 24 h. Each experiment was performed in triplicate.

### Cell proliferation assays

Cell proliferation was determined using the CCK-8 Kit (Dojindo Laboratories, Kumamoto, Japan) according to manufacturer protocols.

### Statistic analysis

All statistical analyses were carried out using the SPSS 17.0 statistical software package (SPSS Inc., Chicago, IL). Rank-sum test was used to analyze the difference between the experiments and controls. All statistical tests were two-sided, *P* < 0.05 was considered to be statistically significant in all cases.
